# The Targeted Maximum Likelihood estimation to estimate the causal effects of the previous tuberculosis treatment in Multidrug-resistant tuberculosis in Sudan

**DOI:** 10.1371/journal.pone.0279976

**Published:** 2023-01-17

**Authors:** Adel Hussein Elduma, Kourosh Holakouie-Naieni, Amir Almasi-Hashiani, Abbas Rahimi Foroushani, Hamdan Mustafa Hamdan Ali, Muatsim Ahmed Mohammed Adam, Asma Elsony, Mohammad Ali Mansournia

**Affiliations:** 1 Department of Epidemiology, National Public Health Laboratory, Khartoum, Sudan; 2 Department of Epidemiology and Biostatistics, School of Public Health, Tehran University of Medical Sciences, Tehran, Iran; 3 Department of Epidemiology, School of Health, Arak University of Medical Sciences, Arak, Iran; 4 Traditional and Complementary Medicine Research Center (TCMRC), Arak University of Medical Sciences, Arak, Iran; 5 Multidrug resistant Tuberculosis Unit- Communicable and Non-Communicable Diseases Control Directorate, Ministry of Health Sudan, Khartoum, Sudan; 6 National Tuberculosis Reference Laboratory, National Public Health Laboratory- Ministry of Health, Khartoum, Sudan; 7 The Epidemiological Laboratory (Epi-Lab), Khartoum, Sudan; Rutgers Biomedical and Health Sciences, UNITED STATES

## Abstract

**Introduction:**

This study used Targeted Maximum Likelihood Estimation (TMLE) as a double robust method to estimate the causal effect of previous tuberculosis treatment history on the occurrence of multidrug-resistant tuberculosis (MDR-TB). TMLE is a method to estimate the marginal statistical parameters in case-control study design. The aim of this study was to estimate the causal effect of the previous tuberculosis treatment on the occurrence of MDR-TB using TMLE in Sudan.

**Method:**

A case-control study design combined with TMLE was used to estimate parameters. Cases were MDR-TB patients and controls were and patients who cured from tuberculosis. The history of previous TB treatment was considered the main exposure, and MDR-TB as an outcome. A designed questionnaire was used to collect a set of covariates including age, time to reach a health facility, number of times stopping treatment, gender, education level, and contact with MDR-TB cases. TMLE method was used to estimate the causal association of parameters. Statistical analysis was carried out with ltmle package in R-software. Result presented in graph and tables.

**Results:**

A total number of 430 cases and 860 controls were included in this study. The estimated risk difference of the previous tuberculosis treatment was (0.189, 95% CI; 0.161, 0.218) with SE 0.014, and p-value (<0.001). In addition, the estimated risk ratio was (16.1, 95% CI; 12.932, 20.001) with SE = 0.014 and p-value (<0.001).

**Conclusion:**

Our findings indicated that previous tuberculosis treatment history was determine as a risk factor for MDR-TB in Sudan. Also, TMLE method can be used to estimate the risk difference and the risk ratio in a case-control study design.

## Introduction

Multidrug resistant tuberculosis (MDR-TB) is defined as the infection with both isoniazid and rifampicin. Globally, there were 132222 reported cases of MDR-TB in 2020. Studies indicated that previous tuberculosis treatment and treatment interruption are considered a main cause of MDR-TB [1, 2]. Epidemiologists define case-control study as a biased sampling design. Case-control study design focuses on parametric logistic regression to calculate adjusted Odd Ratio (OR) conditional on a set of covariates. However, in order to build a causal estimate marginal population, OR should be estimated. Epidemiologists define case control as a biased sampling design of the proportion of those who have diseases compared to the target population. Case-control study design focuses on parametric logistic regression to calculate the OR conditional on a set of covariates. To build a causal estimate we have to estimate the marginal population OR [3].

The targeted maximum likelihood estimation (TMLE) is a double robust methodology uses machine learning algorithm to minimize the risk of bias [4]. Inverse probability treatment weighting (IPTW) is a causal method uses to adjust time-varying confounders by creating similar groups that examine the effect of the treatment on the exposure. IPTW method is based on the probability of exposure given the confounder which is known as propensity score (SP) [5]. IPTW has many drawbacks in case-control studies such that estimators cannot make any claims about asymptomatic efficiency and efficiency issues in finite samples. Also, IPTW has disadvantage the so-called positivity violation which happened when treatment or exposure group are very rare in certain strata define by set of covariates [6]. Thus, case-control weighted TMLE (CCW-TMLE) method provides double robust methods to estimate an unbiased parameters estimation. This method is consistent if any expected parameters from outcome model given exposure and covariates or the exposure model given covariate are correct [7]. CCW-TMLE requires knowledge of the prevalence probability of the outcome to reduce the design biased [8]. In addition, CCW-TMLE estimate various parameters such as risk ratio and risk difference which not available in traditional analysis of case-control study. In addition, TMLE can estimate marginal causal effects, correct specification and propensity score. The TMLE estimates all parameters with assumption that the exposure status of each individual does not affect the potential outcome of any other individuals. The main causal assumption is that there are no unmeasured confounders; therefore, common causes of exposure and outcome have been measured [9].

There are two broad approaches to control confounding during analysis. The first approach is to use standard regression model and the second one is to follow a causal method. Standard regression model not able to estimate the average causal effect of the exposure in the presence of possible confounding or interaction between exposure and covariates. The reason is that, this method assumes no interaction between exposure and confounders to estimate the pool effect. What’s more, the standard regression model fails to adjust for time-varying confounding. Also, the logistic regression model in the presence of time-varying confounders influenced by previous treatment can lead to over-adjustment and collider stratification bias [10–12].

The *ltmle* (longitudinal targeted maximum likelihood estimation) is statistical double robust method can be used to estimate the causal association of the outcome regression. The advantage of TMLE method is that it can report risk ratio and risk difference of exposure in case-control studies, providing a robust estimate of parameters. In addition, the censoring mechanism based on the influence curve enables ltmle to estimate the standard errors and confidence intervals. *ltmle* can incorporate machine learning algorithms to provide a double robust estimator [12]. MDR-TB has been reported in many studies in Sudan. Approximately, 19% of tuberculosis cases were classified as retreated patients while1.8% of them developed MDR-TB [13]. The aim of this study was to estimate the causal association between previous tuberculosis treatment and the occurrence of MDR-TB using TMLE in Sudan.

## Material and methods

The study population consists of Multidrug resistant tuberculosis patients and tuberculosis patients who were susceptible to anti-tuberculosis drugs. The sample size was calculated according to the two-proportion equation where the null hypothesis is that proportion one (p1) equal proportion two (p2). The equation for the sample size calculation was n = ([r+1]/2)*(Za/2+Zb)2*p(1-p)/(p1-p2)2; where p = (p1+p2)/2 (p1 is the exposure among cases and p2 the exposure among control groups. Alpha (Zα2) was calculated as 0.050 (2-sided), the power (Zβ) equaled 0.900, and r was defined as the ratio of controls to cases. Inclusion criteria for the cases were resistant to rifampin and isoniazid and laboratory confirmation. Extrapulmonary and cases died before the commence of the study were excluded from the study. Controls were selected from cured TB patients.

### Data collection

A designed questionnaire was used to collect data from study participants who met the inclusion criteria. The questionnaire was paper-based, validated and piloted prior to data collection. Data was collected by trained interviewers. The study began in May 2017 and was completed in February 2019. Taking previous TB treatment was considered the main exposure variable (A = 1) versus no previous history of TB treatment (A = 0). The outcome (Y = 1) was a patient infected with MDR-TB, (Y = 0) for cured tuberculosis patient, and (W) for possible confounder. Six covariates (w1 to w6) including age, time to reach health facility, number of times stop tuberculosis treatment, gender, education level, and contact with MDR-TB were determined. Other confounders of the MDR-TB include HIV [14], malnutrition, and comorbidities [15]. We assumed that history of previous TB treatment was independent from the occurrence of MDR-TB while controlling for other confounders (w) to achieve conditional exchangeability between exposure and outcome. This means that the risk of getting MDR-TB due exposure was the same as for unexposed. Covariates included in the analysis were significantly associated with MDR-TB in a previous case control study [16].

**Fig 1:**
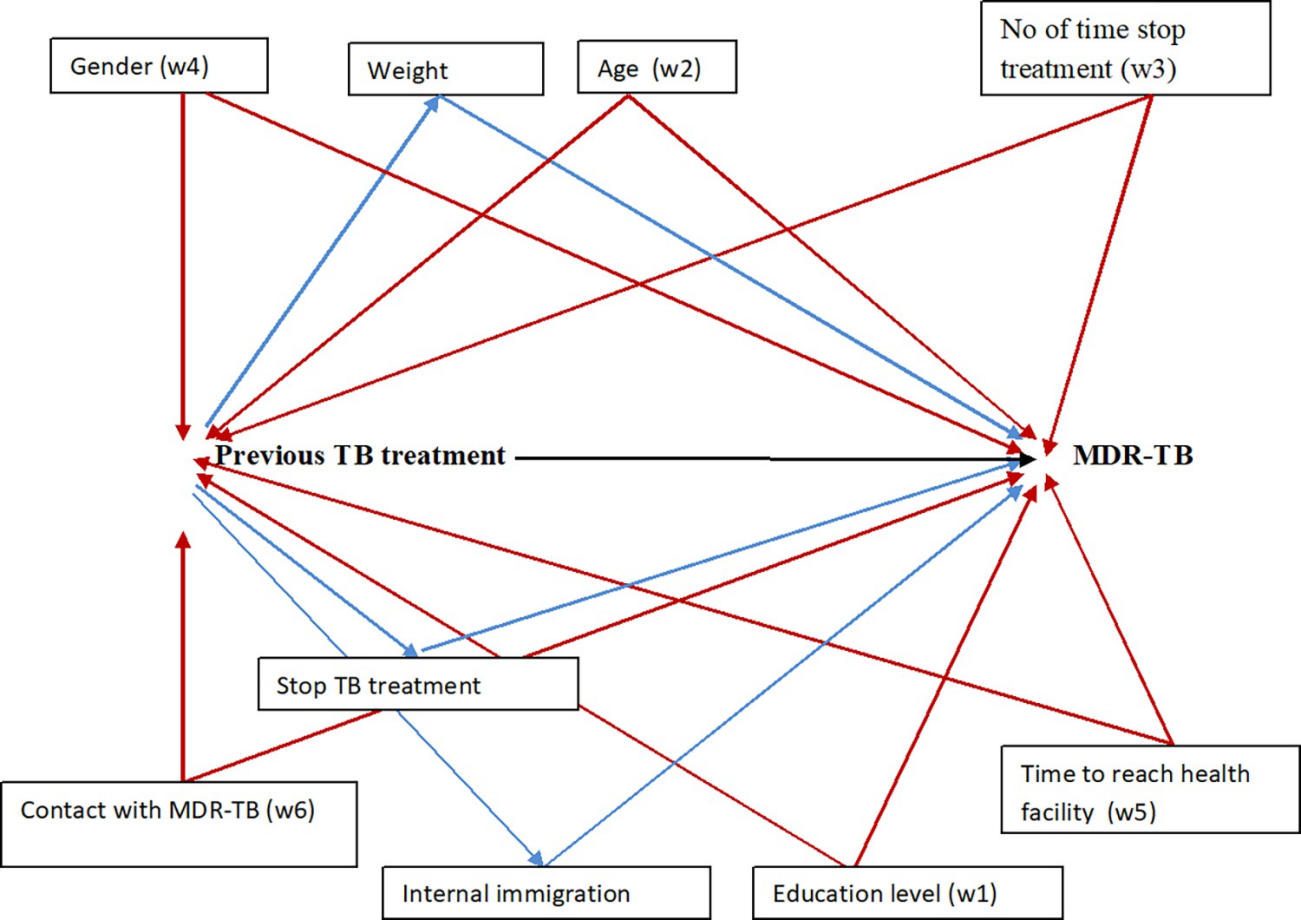
Direct acyclic graph (DAG) showing possible confounders in the association between previous tuberculosis treatment and Multidrug-resistant tuberculosis.

Also, we assume that in the confounders (w) stratum, each subject had a non-zero probability for both exposure conditions. In addition, there was consistency in the observation of possible outcome corresponding to the observed exposure for each study participants. With all these assumptions, we can interpret our findings as a marginal estimation. We used case control weighted TMLE which requires knowledge of the prevalence of the outcome from an external source, here we used Sudan TB data. In the ltmle package, we considered the prevalence of the outcome (Y = 1), which is (0.029), as a weighting number added in the calculation process. Use of prevalence probability help to eliminate bias in case control sampling design [8]. Proper specification of a regression model requires inclusion of minimum sufficient set of confounders, selection of appropriate scale for continuous variables, and inclusion of interaction terms between predictor variables (exposure and confounders). The choice of these confounders was based on the literature and based on the result of the multiple logistic regression. The causal directed acyclic graph (causal diagrams or DAG) was used to determine a set of possible confounding variables which was included in the model (Fig 1) [17]. Direct acyclic graph (DAG) to determine minimal sufficient set (MMS) of variables that will include in the model are; Age, time to reach health facility, number of times TB treatment was stopped”, gender, education level, contact with MDR-TB.

### Statistical methods

The causal method can solve these problems, and it includes three main causal approaches; inverse-probability-of-treatment weighting (IPTW), parametric g-formula, and TMLE, which incorporated these two methods.

### Inverse-Probability-of-Treatment-Weighting (IPTW)

IPTW is based on propensity score (PS), which is the probability of exposure given the confounders. In IPTW, the weights of exposed group is 1/PS, and the weight of unexposed group is 1/ (1-PS) for the. The IPTW has the following steps:

Step 1:

Fit the exposure model either with the regression model of exposure on confounders using logistic regression or be use more advanced methods such as super learners.

Step 2:

Calculate the weight variable (W) from the exposure model fitted in step 1 as follows:

W = 1/PS for the exposed group and W = 1/ (1 –PS) for the unexposed group

Step 3:

Calculate the weighted mean outcome (e.g. risk) in the exposed and unexposed groups using weights (W) which was calculated in step 2, and then calculate the effect measure of interest e.g., risk difference or risk ratio.

IPTW develops a pseudo-cohort, in which confounders do not predict the exposure and the causal effects are the same as in the real cohort. So, the unadjusted (crude) analysis, which is equivalent to the weighted analysis in real cohort, yields an unbiased estimate of the exposure effect assuming that there are no unmeasured confounders and PS does not equal 0 or 1 [18]. Since the PSs are unknown and should be estimated, the IPTW is based on the exposure modeling, For instance, the exposure is regressed on the confounders and then PS and the weights are estimated as mentioned in step 1. Thus, IPTW is recommended when the exposure mechanism is known. The limitation of IPTW is that its sensitive to huge weights of strong predictors of exposure among confounders, which can introduce inefficiency and small sample bias [19].

#### Parametric g-formula

The parametric g-formula is based on standard outcome regression modeling and standardization which is also known as model-based standardization [20]. It has the following steps:

Step1:

Fit the regression model of outcome on the exposure and confounders. Logistic regression or more advanced methods such as super learner can be used.

Step 2:

Calculate the standardized mean outcome in the exposed group (A = 1 where A denote the exposure status taking (1) for the exposed and (0) for the unexposed groups by predicting the individual mean outcome for exposure (1) for all individuals, and actual values of confounders, and then average them over the individuals from the model fitted in step1. Likewise, calculate the standardized mean outcome in the unexposed group (A = 0) by predicting the individual mean outcome for exposure (0) for all individuals, and actual values of confounders, and then average them over the individuals from the model fitted in step 1. Then, calculate the effect measure of interest such as risk difference or risk ratio [7].

### Targeted maximum likelihood estimation

The TMLE is a combination between IPTW, and Parametric g-Formula to produce a double robust causal estimation, which has two possibilities to adjust for confounders. This method provides an unbiased estimate of the causal effect if either exposure or outcome model is correct [21].

### The TMLE has the following steps

First, outcome regression model on the exposure and confounders by using logistic regression for the treatment history Then we used exposure model which is the regression model of exposure on confounders variables. We calculate the variable H from the exposure model We refit the outcome regression model from step 1 by adding the variable H so that the coefficients of the model do not change. We refit the outcome regression model from step 1 by adding the variable H so that the coefficients of the model do not change. We calculate the standardized mean outcome (risk) in the exposed group as (A = 1). Similarly, calculate the standardized mean outcome (e.g., risk) in the unexposed group (A = 0).

If any of outcome regression model or exposure regression model is correct, then TMLE gives an unbiased estimates of the causal effects of the treatment history [22]. Confidence intervals can be calculated using the influence function (IF).

### Model specification

There are several modeling approaches that can be used to build relationship between variables such as neural network, Bayesian methods, classification and regression trees, random forests, generalized boosted regression, and generalized additive models. A machine learning approach, which is called Super Learner (SL) is a powerful method for the modeling of exposure and outcome in the causal inference. The super learner is a combination of several regression models such as logistic regression, neural network, Bayesian approach, classification and regression trees, random forests, generalized boosted regression, generalized additive models, etc. In super learner method, a best weight for each model can obtain by the mean of square difference between observed value and the predicted value of the outcome [23].

For the analysis, we used the newly developed ltmle R package, which is the targeted maximum likelihood estimation (TMLE) for estimating parameters for both point estimate and longitudinal data. ltmle apply estimator which can able to estimate ATE, causal risk ratio, and causal odds ratio. This package is flexible and can be applied in our study design as case control [24]. First, we specified the structural causal model (SCM) in order to encode knowledge on covariates relationship. Two important assumptions that allow identification of the distribution of our causal model are the sequential randomization and positivity assumption. Under these two assumptions, the counterfactual model can be identified by the longitudinal G-computation formula. The first assumption was that no unmeasured confounders exist, while the second assumption was that each observation has a positive probability.

In addition to additive effect, risk ratio and odds ratio, ltmle function provides estimate for mean intervention. Our data was entered into R software by formulating a data frame, which is working platform for the ltmle package to work. Since our data was a case-control study, we identified treatment (exposure) as node (A) which is defined by Anodes, and the outcome node (Y) specified with Y in the ltmle package. We didn’t have time varying variable so we put Null for L nodes. We specified (0,1) for the abar (binary vector), and assigned weight to our data sample by using observation-weights = as vector (Wt) function. We used two regression formula, a character vector Qform, and qfrom, where our regression model including outcome (Y), exposure (A) and set of covariates were added. We put iptw as FLASE which led to calculate both TMLE and IPTW. We used the variance method “ic” which computed a robust variance estimator using TMLE and the influence curve based variance estimator [25]. Qfrom and qfrom were working as a design amatrix which allowed calling the Super Learner (SL).

We used the Super Learner algorithm to estimate the component of Q and q by implementing (SL) package. The SL algorithm chooses the best weighted estimator through a validation process. The SL has a lower bias compared to single parametric model and consistent when we estimate the Q and q components [26]. Estimating both Q and q was provided a consistent TMLE, which achieved the best possible variance. When SL was used, all variables on the right side of the formula were considered predictors in the regression model. Using ltmle provides a summary that returns the estimator‘s parameter values, estimated variance, 95% confidence interval, and p.value. Summary of ltmle returned also the additive treatment effect for each of the two objects in the abar list. Moreover, relative risk and odds ratio were calculated along with their variance, confidence interval, and p-value [27]. We used R software (version 3.5.3) for data analysis and this software used super learner to provide valid statistical inference [28].

## Result

A total number of 430 cases and 860 controls with mean age 35.3 ± 13.17 for cases and 38.27± 16.12 for controls were included in this study. Study participants consist of 896 males (295 cases, 601 controls) and 374 females (130 cases, 294 controls). Participants with primary and secondary education were 27.1% and 27.6% respectively. Participants without education represented 19.5% of the study population. A total 292 (67.9%) of cases had history of previous TB treatment while only 26 (3.0%) of the control had received previous TB treatment (Table 1). Previous TB treatment history was included as exposure variable, cases and controls as outcome variables, and a set of confounders were included in the analysis. These confounders included age, time to reach health facility, Number of times stop treatment, gender, education level, and contact with MDR-TB. Summary of ltmle returns treatment (exposure in our setting) estimator and the parameter estimate was (0.202, 95% CI; 0.174, 0.23009) with standard error (0.014309) and p.value (<0.001). Also, the estimator and parameter estimate of the control group was (0.012562, 95% CI; 0.010456, 0.014669) with standard error (0.0010748) and p. value (<0.001). The causal risk difference estimate was (0.18948, 95% CI; 0.16135, 0.21761) with SE 0.014353, and p. value (<0.001). Moreover, the causal Risk ratio was 21(16.083, 95% CI; 12.932, 20.001) with SE equal 0.014353 and p. value (<0.001) (Table 2). This result suggested that the risk among exposed was 16 times compared with the risk of unexposed. Patients who had been exposed to previous TB treatment history were 18% more likely develop MDR-TB compared with patients who had not exposed to tb treatment. Narrow confidence interval for the estimated parameters indicates an strong statistical association.

**Table 1:** Demographic and clinical characteristics of stud participants.

	Cases (430)	Control (860)	Total (1290)
**Age**			
Mean	35.31	38.25	
SD	13.17	16.12	
**Gender**			
Male	30 (69.7%)	606 (70.4%)	906
Female	130 (30.3%)	254 (29.6%)	384
**Education level**			
Without education	60 (14.1%)	192 (22.3%)	252
Khalwa	39 (9.2%)	55 (6.4%)	94
Primary	129 (29.9%)	220 (25.6%)	349
Secondary	117 (26.8%)	240 (27.9%)	357
Diploma	8 (1.9%)	1 (0.1%)	9
University + postgraduate	77 (18.1%)	152 (17.7%)	229
**Time to reach health center (hs)**			
Mean	2.2	1.9	
Median	1.2	1.5	
SD	4.4	1.2	
**Number of family member (per room)**			
Mean	7.5	8.3	
Median	7	8	
SD	3.6	3.6	
**Contact with TB patients**			
Yes	97 (22.6%)	58 (6.8%)	155
No	309 (71.9%)	783 (91.0%)	1077
I don’t remember	24 (5.5%)	19 (2.2%)	43
**Contact with MDR-TB patients**			
Yes	64 (14.9%)	2 (0.2%)	66
No	339 (78.8%)	844 (98.2%)	1183
I don’t remember	27 (6.3%)	14 (1.6%)	41
**History of previous treatment**			
Yes	292 (67.9%)	26 (3.0%)	318
No	138 (32.1%)	834 (97.0%)	972
**Diabetes**			
Yes	30 (7.0%)	46 (5.3%)	76
No	400 (93.0%)	814 (94.7%)	1214
**Fever**			
Yes	242 (56.3%)	379 (55.9%)	723
No	188 (43.7%)	370 (44.1%)	567

**Table 2:** Measurement of Targeted Maximum Likelihood Estimation (TMLE).

Measurement	Estimate (95% CI)	Standard error	p- value
**Risk difference**	0.189 (0.16135, 0.21761)	0.014353	<0.001
**Risk Ratio**	16.1 (12.93, 20.00)	0.014353	**0000** <0.001

## Discussion

In this study, we implemented a double robust method to estimate the causal effect of parameters in a case-control study. We implemented TMLE as a robust method to estimate the causal effect of the previous TB treatment history on the occurrence of the MDR-TB in Sudan. Our main finding was that the previous TB treatment was identifies as a risk factor for MDR-TB. We used TMLE as statistical method to eliminate the effect of confounding variables that can affect parameters estimation in the traditional case-control study [29]. In observational studies, such as cross-sectional studies and case-control studies, we cannot establish a causal association between exposure and outcome.

In traditional case-control study it is not able to estimate the risk difference and the risk ratio. Using ordinary logistic regression depends on the selection algorithms, such as stepwise regression, which can result in missing very important confounders. Also, logistic regression reports only the Odds ratio for the association between the exposure and the outcome. To establish causal effect association, risk ratio and risk difference need to be estimated. Thus, R ltmle package is able to estimate these causal effects association of parameters using double robust method. Therefore, we applied ltmle in this study to establish a causal association the previous TB treatment (exposure) and the development of the MDR-TB (outcome). TMLE was used by study conducted to model the treatment effect of MDR-TB patient data metanalysis. In this study targeted maximum likelihood estimate was used to provide robust estimation of parameters [30]. TMLE provided a precise estimation for the causal parameters and less prone to bias compared to other estimators such as inverse probability weighting estimator (IPW) and propensity score [31].

The advantage of using such method can help researcher to establish a causal association for observational studies. Compared with ordinary logistic regression, with TMLE can remove all biases due to model misspecification [32]. TMLE was also used by a group of researchers to assess the effect of MDR-TB outcome in 31 observational studies We have shown in this study a successful implementation of ltmle in a case-control study design using an original data set [33]. Our Findings show that bias can be eliminated by using machine learner algorithms through the Super Leaner package in ltmle. Also, our study provided an evidence that the ltmle can be considered as a general template for building an efficient estimator for the targeted parameters [34].

The result of study providing a guide for people who are interested in TB and MDR-TB to conduct similar studies in their setting. Many studies reported that tuberculosis treatment interruption is the main cause of MDR-TB. In study conducted in Bangladesh, previous TB treatment was associated with the MDR-TB [35]. In a systematic review conducted in Ethiopia, the burden of the MDR-TB was high in Ethiopian setting particularly among previously treated TB patents [36]. In study conducted in Vietnam among TB patients indicated that an increase in the number of MDR-TB cases and previous TB treatment are the greatest risk of poor treatment outcome [37].

Using TMLE as statistical tool estimate risk ratio and risk difference was providing an evidence to scientists that previous treatment is a risk for the MDR-TB occurrence. Many researchers have interest to conduct observational studies, so this study provided an evidence that TMLE, as a doubled robust method, can be applied in traditional case-control study design. The existence of R Software as a free source will help researchers, particularly those in countries with limited resources, to apply TMLE in their research. In addition, further researches are needed to study the risk factors associated with MDR-TB, particularly in countries with a high burden of TB. One limitation of using TMLE is that the estimation of variance is challenging when the positivity assumption is violated. Secondly, in a rare binary outcome, to estimate the outcome models in the existence of a vector of covariates can produce a bias variance in moderate sample size. In this study we demonstrated the use of prevalence probability in weighted case-control using targeted maximum likelihood estimation to obtain efficient causal parameter. We also able to estimate risk ratio and risk difference in a traditional case-control study design. Moreover, previous history of TB treatment was identifies as a risk factor for the occurrence of MDR-TB in Sudan.

## Supporting information

S1 File (CSV)
