# Correction: The Targeted Maximum Likelihood estimation to estimate the causal effects of the previous tuberculosis treatment in Multidrug-resistant tuberculosis in Sudan

**DOI:** 10.1371/journal.pone.0314954

**Published:** 2024-12-02

**Authors:** Adel Hussein Elduma, Kourosh Holakouie-Naieni, Amir Almasi-Hashiani, Abbas Rahimi Foroushani, Hamdan Mustafa Hamdan Ali, Muatsim Ahmed Mohammed Adam, Asma Elsony, Mohammad Ali Mansournia

The eighth author’s initials are indexed incorrectly in PubMed. The correct initials are: Mansournia MA.
